# Dr. William H. Hoopes

**Published:** 1895-02

**Authors:** 


### Obituary.

### DR. WILLIAM H. HOOPES.

DR. WILLIAM H. HOOPES died January 30th, 1895. He was born January 18th, 1835 in Chester County, Pennsylvania, and was a decendant of Joshua Hoopes, who came from England in 1683. His father moved to Maryland and Dr. Hoopes at the age of seventeen, entered the office of Drs. Townsend and Chandlee, graduated from Baltimore College of Dental Surgery in 1856 and immediately began practice of his profession in Baltimore, which he continued until his last illness. Dr. Hoopes was a very skillful dentist, and especially in the practice of dental prosthesis acquired a well merited reputation. The successful construction of a combined artificial nose, lip and obturator by Dr. Hoopes in 1860, in a case where there was a terrible destruction of the bones and soft parts of the face, and which was fully described in the AMERICAN JOURNAL OF DENTAL SCIENCE of the same year, was regarded as a very skillful operation of nasal and maxillary prosthesis, and was very widely commented upon.

He was a trustee of the Maryland Academy of Science and was also interested in several other societies.

